# Plasma-derived exosomal mRNA profiles associated with type 1 diabetes mellitus

**DOI:** 10.3389/fimmu.2022.995610

**Published:** 2022-09-13

**Authors:** Wenqi Fan, Haipeng Pang, Xiajie Shi, Jiaqi Li, Yimeng Wang, Shuoming Luo, Jian Lin, Haibo Yu, Yang Xiao, Xia Li, Gan Huang, Zhiguo Xie, Zhiguang Zhou

**Affiliations:** ^1^ National Clinical Research Center for Metabolic Diseases, Key Laboratory of Diabetes Immunology (Central South University), Ministry of Education, Changsha, China; ^2^ Department of Metabolism and Endocrinology, The Second Xiangya Hospital of Central South University, Changsha, China

**Keywords:** type 1 diabetes mellitus, exosomes, messenger RNAs, biomarker, plasma

## Abstract

**Background:**

Exosomes carry various types of transcripts, such as messenger RNAs (mRNAs), and play an important role in mediating cell-to-cell communication, thus influencing multiple physiological and pathological processes. However, the role of exosomal mRNAs in T1DM is largely unknown. Here, we aimed to identify the plasma-derived exosomal mRNA expression profiles in T1DM and to explore their potential biological functions in T1DM.

**Materials and Methods:**

Plasma-derived exosomes were isolated from 10 patients with T1DM and 10 age- and sex-matched control subjects by size exclusion chromatography methods. Transmission electron microscopy, nanoparticle tracking analysis, and western blot analysis confirmed the presence of exosomes. The exosomal mRNAs were analyzed using the Illumina HiSeq platform. Six differentially expressed mRNAs (DEMs) were randomly selected to determine the expression level by quantitative real-time PCR (qRT−PCR) in a larger cohort (T1DM subjects N=40; control subjects N=40). The biological functions of DEMs were predicted by Gene Ontology (GO) and Kyoto Encyclopedia of Genes and Genomes (KEGG) analyses. Protein−protein interaction networks were constructed to explore the potential associations among DEMs.

**Results:**

In total, 112 DEMs were identified in T1DM, among which 66 mRNAs were upregulated and 46 mRNAs were downregulated. Four of six candidate exosomal mRNAs were successfully validated by qRT−PCR. Bioinformatics analysis indicated that these mRNAs were most significantly involved in positive regulation by host viral transcription (GO enrichment analysis) and oxidative phosphorylation (KEGG pathway analysis).

**Conclusions:**

Our study reported the plasma-derived exosomal mRNA expression profiles of T1DM for the first time. The identified DEMs might be associated with the pathogenesis of T1DM, and some DEMs have the potential to serve as biomarkers and therapeutic targets for T1DM.

## Introduction

Type 1 diabetes mellitus (T1DM) is defined as a chronic disease caused by autoimmune attack against pancreatic islet beta-cells (1). At present, most patients with T1DM have to rely on lifelong insulin replacement therapy. It has been indicated that the incidence of T1DM peaks at the ages of 10-14 years, and the estimated number of children and adolescents with T1DM is increasing worldwide (2). In addition, because of long-standing hyperglycemia, diabetes mellitus will induce severe chronic injury and dysfunction in all kinds of tissues and organs, imposing tremendous health and economic burdens on patients. It has been widely accepted that the pathophysiological process of T1DM is induced by environmental factors in individuals who are at high genetic risk (3, 4). However, many aspects of the pathogenic process of T1DM are unknown.

Recently, a small membrane-derived lipid bilayer vesicle, namely, the exosome (30-200 nm in diameter), has been shown to be of great importance in mediating intercellular and interorgan communication (5, 6). The bioactive materials delivered by exosomes, such as DNA, RNA (mRNA, lncRNA, and miRNA) and proteins, can be transferred into recipient cells and can alter their status and function (7, 8). In the context of T1DM, the abnormal interactions between pancreatic beta-cells and immune cells, especially T lymphocytes, represent the main pathogenic mechanism (9). Interestingly, mounting evidence has suggested that exosomes might serve as novel mediators between beta-cells and immune cells. For instance, islet-derived exosomes contain beta-cell autoantigens and can be taken up by dendritic cells, thus leading to cell activation (10). In addition, exosome release by T lymphocytes can induce beta-cell apoptosis *via* certain exosomal miRNA-mediated signaling (11). In addition, given that the content of exosomes can reflect the status of the originating cells, exosomes are viewed as promising diagnostic tools for many diseases. It has been indicated that human islet-derived exosomal RNAs are dysregulated under cytokine stress (12). Our recent study characterized the lncRNA profiles of plasma-derived exosomes from T1DM patients and explored their potential biomarker use (13). In addition, a study measured plasma-derived exosomal miRNA expression profiles and reported a distinct miRNA signature in long-duration T1DM (14), which highlighted the biomarker potential of exosomes in T1DM.

Existing studies have mostly focused on exosomal miRNAs. Here, we report the mRNA expression profiles of plasma-derived exosomes in T1DM for the first time. Our work might provide novel insights into the pathogenesis of T1DM and lay the foundation for the use of exosomal mRNA as a biomarker.

## Materials and methods

### Study subjects

This study was approved by the institutional ethics review board of the Second Xiangya Hospital of Central South University, and all experiments complied with the ethical principles of the Declaration of Helsinki. The inclusion and exclusion criteria of T1DM patients and control subjects have been described previously (13). In the discovery phase, 10 cases and 10 age- (*P*=0.732) and sex- (*P*=0.650) matched controls were recruited (13). We used a larger cohort (T1DM N=40; controls N=40) for the subsequent validation phase. All peripheral blood samples were obtained after full informed consent was received.

### Isolation and characterization of exosomes

Peripheral blood samples (5 ml) from participants were collected in EDTA tubes. The plasma was separated and stored at -80°C after centrifugation at 3000×g for 15 min at 4°C. Exosomes were isolated by using size exclusion chromatography with Exosupur^®^ columns (Echobiotech, China) and were characterized by transmission electron microscopy (TEM), nanoparticle tracking analysis (NTA), and western blotting (WB) (performed by EchoBio Technology, Beijing, China). The detailed procedures have been described previously (13). In brief, the filtered plasma was diluted with phosphate-buffered saline and purified by Exosupur^®^ columns. The collected fraction was concentrated by a 100-kDa molecular weight cutoff Amicon^®^ Ultra spin filter (Merck, Germany) to obtain exosomes. Exosomes (10 µL) were placed on a copper mesh and negatively stained with uranyl acetate solution. Then, the sample was examined by TEM (H7650, Hitachi Ltd, Tokyo, Japan). The size distribution of isolated exosomes was determined by a ZetaView PMX 110 (Particle Metrix, Meerbusch, Germany) and analyzed by NTA software (ZetaView 8.02.28). Finally, the exosomes were subjected to WB analysis using rabbit polyclonal antibodies against TSG101, Alix, CD63 and calnexin. The verification of exosomes was entrusted to the company (Echo Biotech Co., Ltd, Beijing, P. R. China).

### RNA extraction and mRNA sequencing

The exosomal RNA was extracted using the miRNeasy Serum/Plasma Advanced Kit (Qiagen, cat. No. 217204). RNA profiles were assessed by using the RNA Nano 6000 Assay Kit of the Agilent Bioanalyzer 2100 System (Agilent Technologies, CA, USA). After library construction and evaluation, sequencing was performed by an Illumina NovaSeq6000 platform (performed by EchoBio Technology, Beijing, China).

### mRNA analysis

Raw reads in fastq format were first processed through in-house Perl scripts. In this step, clean reads were obtained by removing reads containing adapters, reads containing poly-N sequences and low-quality reads from the raw data. At the same time, the Q20, Q30, GC content and sequence duplication level of the clean data were calculated. All downstream analyses were based on clean data with high quality. Paired-end clean reads were aligned to the reference genome GRCh38 using HISAT2. Mapped reads were used for the quantification of gene expression levels and differential expression analysis. StringTie was used to explore novel mRNAs and to calculate FPKMs (reads per kilobase per million mapped reads) of coding genes in each sample. Gene FPKMs were computed by summing the FPKMs of transcripts in each gene group. Sequencing data analyses were mainly performed using R v3.5.1. We applied the Mann−Whitney U test to carry out the differential expression analysis with the cutoffs of FPKM > 5, *P* value < 0.05 and |log2(FC)| > 0.584. Heatmaps, volcano diagrams, and MA plots were generated to visualize the differentially expressed mRNAs (DEMs) using the R packages “pheatmap” and “ggplot2”.

### Functional enrichment analysis of identified mRNAs

After the completion of mRNA analysis, BLAST software was adopted to compare the new genes with the NR, SwissProt, GO, COG, and KEGG databases to obtain annotation information, and then Gene Ontology (GO) and Kyoto Encyclopedia of Genes and Genomes (KEGG) pathway enrichment analyses were performed by using the topGO R packages and KOBAS software to explore the potential functions of the identified exosomal DEMs (15).

### Protein−protein interaction network

The Search Tool for the Retrieval of Interacting Genes (STRING) (v11.5) was used to predict the associations among DEMs. A combined score > 0.4 was considered a statistically significant interaction. A protein‐protein interaction (PPI) network was constructed using Cytoscape v3.7.0. Using the CytoHubba plug-in of Cytoscape software, each gene was scored based on the MNC, DMNC, MCC, EPC, and degree algorithms. Based on the scoring results of the five algorithms, the top 8 genes in each algorithm were considered hub genes.

### Quantitative real-time PCR analysis

To validate the expression level of DEMs, six mRNAs, namely, ENSG00000158417, ENSG00000185883, ENSG00000198763, ENSG00000198786, ENSG00000198840 and ENSG00000269028, were randomly selected to perform qRT−PCR analysis in an independent cohort including 40 T1DM and 40 control subjects. The detailed information of the participants is summarized in **Table 1**. All T1DM patients were at a symptomatic stage, characterized by the presence of islet autoantibodies and persistent hyperglycemia. GAPDH was used as an internal reference. Briefly, total RNA was extracted from plasma-derived exosomes and then reverse transcribed into cDNA by using the PrimeScript™ RT reagent Kit (Perfect Real Time) (TAKARA, RR037A). The expression level of candidate mRNAs was measured with the TaqMan^®^ probe using qPCR, and all experiments were performed in triplicate wells. The primers and probes are shown in **Table 2**. We used an unpaired t test to compare the expression levels of selected mRNAs between the two groups. A *P* value < 0.05 was considered statistically significant. The results were visualized using the R package.

**Table 1:** Detailed information of T1DM patients and control subjects in the validation phase.

Characteristics	T1DM (n=40)	Control (n=40)	*P* value
Sex (male/female)	17/23	19/21	0.65
Age (years)	27.35 ± 8.68	28.4 ± 5.00	0.51
Duration (months)	25.76 ± 14.37	NA	NA
FPG (mmol/L)	7.79 ± 3.25	4.75 ± 0.40	0.00
HbA1c %	7.55 ± 2.04	5.37 ± 0.23	0.00
TC (mmol/L)	4.26 ± 0.83	4.28 ± 0.46	0.90
HDL (mmol/L)	1.59 ± 0.43	1.31 ± 0.39	0.01
LDL (mmol/L)	2.36 ± 0.70	2.49 ± 0.42	0.34
TG (mmol/L)	0.76 ± 0.30	0.90 ± 0.32	0.08
CREA (μmol/L)	63.07 ± 12.53	66.21 ± 14.75	0.36
Age of onset (years)	25.60 ± 8.13	NA	NA
FCP (pmol/L)	94.40 (39.10-148.40)	NA	NA
2 h-PCP (pmol/L)	157.05 (88.93-466.45)	NA	NA
GADA positivity (%)	90.00	NA	NA
GADA titer (u/ml)	149.25 (27.73-575.88)	NA	NA
IA-2A positivity (%)	55.00	NA	NA
IA-2A titer (u/ml)	17.79 (0-800.52)	NA	NA
ZnT8A positivity (%)	35.00	NA	NA

FPG, fasting plasma glucose; HbA1c, hemoglobin A1c; TC, total cholesterol; LDL, low-density lipoprotein; CREA, creatinine; FCP, fasting C-peptide; PCP, postprandial C-peptide; GADA, glutamic acid decarboxylase antibody; IA-2A, protein tyrosine phosphatase antibody; ZnT8A, zinc transporter 8 antibody; NA, not applicable.

**Table 2:** Primer list.

mRNA	Symbol	Primer Sequence (5’-3’)
ENSG00000158417	EIF5B	F-GATGGTGAAGCAGGTGGTAT
		R-CCTTTGCTCGCTCCTCAA
		P-CCTGACTCTGATGTGGCTGCTACTTT
ENSG00000185883	ATP6V0C	F-Forward-CTCATCTTCGCCGAGGTG
		R-CGTTCTGGAATGAGGAGGG
		P-CGAGCCCACCAGCCACAGAA
ENSG00000198763	MT-ND2	F-ACTCAACTTAAACTCCAGCACC
		R-TAGGCGTAGGTAGAAGTAGAGG
		P-ATCTCGCACCTGAAACAAGCTAACA
ENSG00000198786	MT-ND5	F-CCTGTAGCATTGTTCGTTACATG
		R-ATGCTAAGGCGAGGATGAAAC
		P-CAACACAGCAGCCATTCAAGCAATC
ENSG00000198840	MT-ND3	F-CCACAACTCAACGGCTACAT
		R-GTTGTTTGTAGGGCTCATGGT
		P-CGAGTGCGGCTTCGACCCT
ENSG00000269028	MTRNR2L12	F-AGATTTATAGGTAGAGGCGACAAAC
		R-ACGATGGGTGTTGAGCTTG
		P-TGGTGATAGCTGGTTGTCCAAGATAGAATC

F-, forward sequence; R-, reverse sequence; P-, probe sequence.

## Results

### Characterization of exosomes

**Figure 1:**
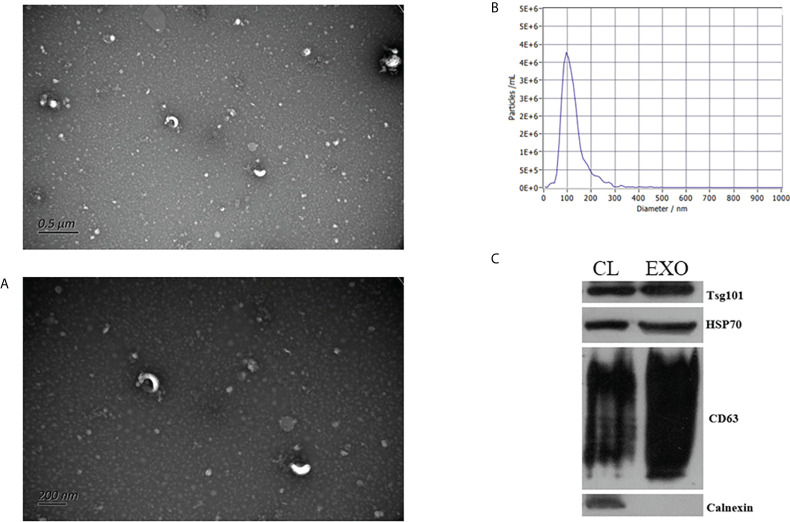
Identification of exosomes. Transmission electron microscopy images of exosomes **(A)**. Nanoparticle tracking analysis results **(B)**. Western blot analysis of exosome marker proteins **(C)**.

To validate the detection of exosomes, we performed TEM, NTA, and WB analysis. As shown in **Figures 1A, B**, the isolated particles were oval- and cup-shaped and were approximately 121.5 nm in diameter (30 nm to 200 nm), consistent with the morphological characteristics of exosomes. With WB analysis, the well-known exosomal marker proteins Alix, Tsg101, and CD63 were detected in isolated fractions, while the negative marker of exosomes, calnexin, was absent (**Figure 1C**). Thus, exosome samples were well prepared and had good purity.

### Plasma-derived exosome mRNA profiling

**Figure 2:**
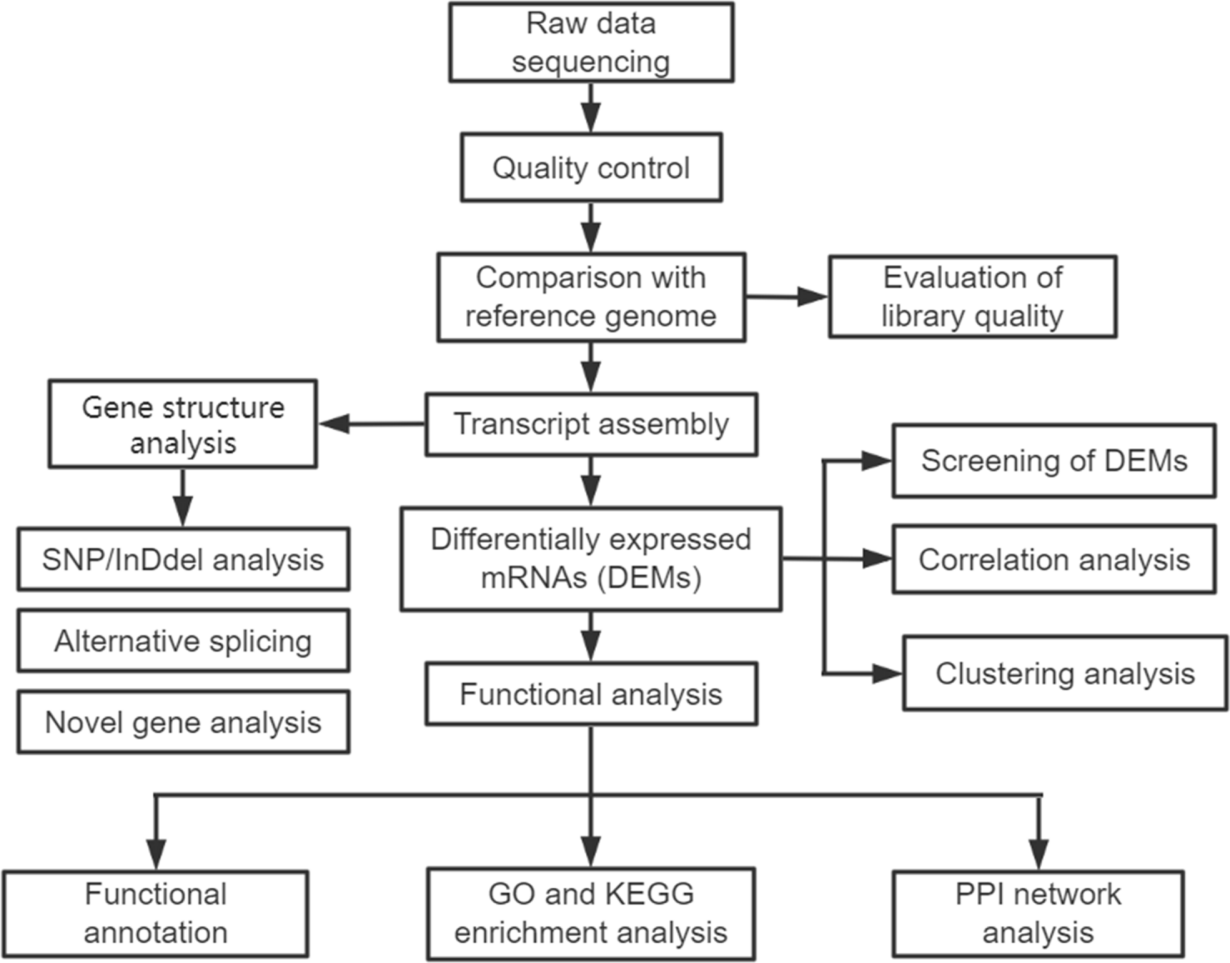
The analytical procedure for the sequencing data of exosomal mRNAs.

The analytical process for the sequencing data is summarized in **Figure 2**. A total of 20 samples, including 10 patients and 10 healthy controls, with 478.89 Gb of clean data were constructed. The clean data of each sample were at least 19.67 Gb. Each sample was compared with the reference genome sequence, and the alignment efficiency ranged from 23.42% to 68.53%. A total of 22,298 mRNAs, including 19,986 known mRNAs and 2312 novel mRNAs, were generated (**Table S1**). The annotation information of new genes was obtained by using BLAST software (**Table S2**).

### Differentially expressed mRNAs

**Figure 3:**
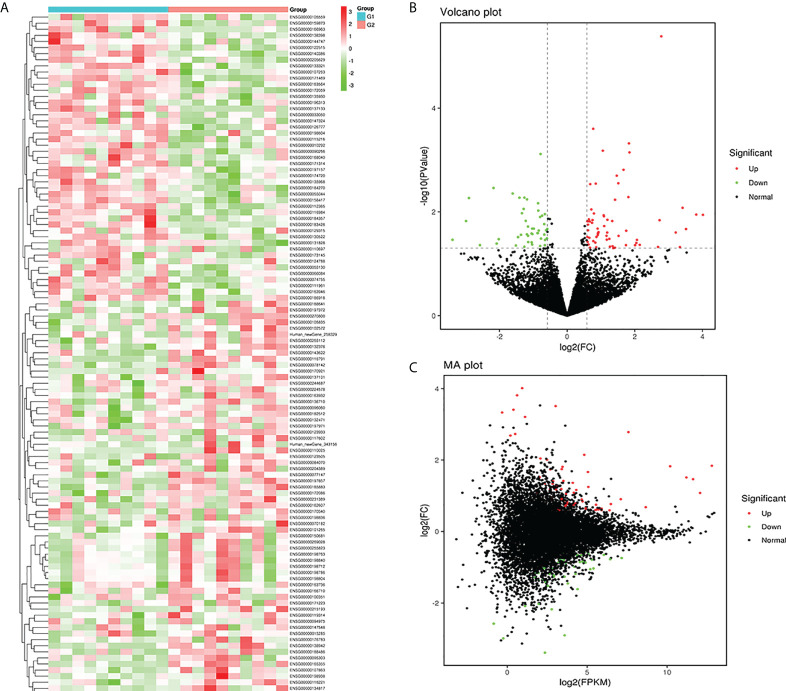
Exosomal mRNA profiles of type 1 diabetes mellitus (T1DM). Heatmap **(A)** of the expression levels of the identified differentially expressed mRNAs (DEMs). Red and green represent high and low expression, respectively. G1: control group (N=10); G2: case group (N=10). A volcano diagram **(B)** and M-versus-A **(C)** plot were used to show DEMs in T1DM. The Mann−Whitney U test was used to identify DEMs with cutoffs of FPKM > 5, *P* value < 0.05 and |log2(FC)| > 0.584. Upregulated DEMs (red dots); downregulated DEMs (green dots); nondifferentially expressed mRNAs (black dots).

In this study, plasma-derived exosome samples from 10 patients with T1DM and 10 healthy controls were used for RNA sequencing. A total of 112 mRNAs were differentially expressed (*P* value < 0.05), of which 66 mRNAs were upregulated and 46 mRNAs were downregulated. We performed hierarchical clustering to sort the identified exosomal DEMs (**Figure 3A**). A volcano diagram was used to intuitively show the relationship between the *P* value and the fold change of all transcripts to quickly view the expression level difference and statistical significance of mRNAs between the two groups of samples (**Figure 3B**). The overall distribution of the expression abundance and fold changes of mRNAs in the two groups is shown through the M-versus-A plot (**Figure 3C**). In addition, all DEMs sorted by *P* values are detailed in **Table 3**. Our results demonstrated that the mRNA expression profiles of plasma-derived exosomes were distinctly different between T1DM patients and controls.

**Table 3:** Detailed information on the DEMs.

Gene ID	Symbol	*P* Value	log2FC	Direction of Regulation
ENSG00000198938	MT-CO3	4.14E-06	2.782842	up
ENSG00000185883	ATP6V0C	0.00025	0.770056	up
ENSG00000198840	MT-ND3	0.000478	1.825964	up
ENSG00000101265	RASSF2	0.00066	1.053995	up
ENSG00000198763	MT-ND2	0.000713	1.841181	up
ENSG00000158417	EIF5B	0.000766	-0.78645	down
ENSG00000269028	MTRNR2L12	0.001543	1.665036	up
ENSG00000198712	MT-CO2	0.002004	1.464607	up
ENSG00000198786	MT-ND5	0.002809	1.512536	up
ENSG00000105835	NAMPT	0.002853	0.844394	up
ENSG00000166710	B2M	0.002921	0.681995	up
ENSG00000171469	ZNF561	0.003456	-2.17733	down
ENSG00000130522	JUND	0.004426	-1.61195	down
ENSG00000255823	MTRNR2L8	0.005186	1.810294	up
ENSG00000159873	CCDC117	0.005312	-1.26336	down
ENSG00000140386	SCAPER	0.005329	-1.41142	down
ENSG00000205629	LCMT1	0.0054	-2.90529	down
ENSG00000055044	NOP58	0.005735	-1.18571	down
ENSG00000095303	PTGS1	0.00584	1.416542	up
ENSG00000135968	GCC2	0.006818	-0.86068	down
ENSG00000138942	RNF185	0.00834	3.411566	up
ENSG00000111961	SASH1	0.008765	-1.2861	down
ENSG00000123505	AMD1	0.008967	0.714045	up
ENSG00000138398	PPIG	0.009431	-0.85431	down
ENSG00000129315	CCNT1	0.010629	-1.33722	down
ENSG00000198836	OPA1	0.010736	0.789939	up
ENSG00000198604	BAZ1A	0.011056	-0.66033	down
ENSG00000165355	FBXO33	0.011263	3.816015	up
ENSG00000116791	CRYZ	0.011406	4.014323	up
ENSG00000198804	MT-CO1	0.011611	1.079629	up
ENSG00000184357	H1-5	0.01183	-0.74095	down
ENSG00000172086	KRCC1	0.012191	1.085618	up
ENSG00000144747	TMF1	0.012538	-0.8008	down
ENSG00000231389	HLA-DPA1	0.013866	0.642742	up
ENSG00000197971	MBP	0.014185	1.189494	up
ENSG00000182512	GLRX5	0.014364	0.750828	up
ENSG00000096060	FKBP5	0.014452	0.747055	up
ENSG00000134817	APLNR	0.014467	2.730512	up
ENSG00000186918	ZNF395	0.014881	-1.22411	down
ENSG00000132376	INPP5K	0.014988	1.332368	up
ENSG00000183426	NPIPA1	0.014994	-2.98966	down
ENSG00000132471	WBP2	0.015164	0.595977	up
ENSG00000102572	STK24	0.015314	0.611512	up
ENSG00000147548	NSD3	0.015318	0.671293	up
ENSG00000117602	RCAN3	0.016233	0.735737	up
ENSG00000163946	TASOR	0.017167	-0.67946	down
ENSG00000170540	ARL6IP1	0.017579	0.614128	up
ENSG00000077147	TM9SF3	0.018746	1.095219	up
ENSG00000116984	MTR	0.01916	-1.15852	down
ENSG00000150681	RGS18	0.020987	0.900095	up
ENSG00000090266	NDUFB2	0.020992	-1.03341	down
ENSG00000168040	FADD	0.021014	-0.98522	down
ENSG00000115216	NRBP1	0.021257	-0.58867	down
Human_newGene_343156	Human_newGene_343156	0.021403	3.513743	up
ENSG00000171314	PGAM1	0.022593	-0.62364	down
ENSG00000084070	SMAP2	0.022629	0.616291	up
ENSG00000174720	LARP7	0.022858	-0.84092	down
ENSG00000171223	JUNB	0.023075	1.745297	up
ENSG00000188641	DPYD	0.024545	0.926448	up
ENSG00000197857	ZNF44	0.024939	3.208025	up
ENSG00000123933	MXD4	0.024984	1.183135	up
ENSG00000131828	PDHA1	0.025616	-0.86785	down
ENSG00000010292	NCAPD2	0.025664	-0.87864	down
ENSG00000163736	PPBP	0.025931	0.904892	up
ENSG00000135930	EIF4E2	0.026074	-1.05251	down
ENSG00000162607	USP1	0.026174	0.913166	up
ENSG00000066084	DIP2B	0.026774	-1.02817	down
ENSG00000173145	NOC3L	0.027063	-1.18953	down
ENSG00000126777	KTN1	0.027356	-0.67727	down
ENSG00000122515	ZMIZ2	0.027625	-1.36426	down
ENSG00000124788	ATXN1	0.028118	0.901701	up
ENSG00000147324	MFHAS1	0.028845	-1.15761	down
ENSG00000224578	HNRNPA1P48	0.028899	1.162777	up
ENSG00000119138	KLF9	0.028975	1.519606	up
Human_newGene_258329	Human_newGene_258329	0.030406	0.594307	up
ENSG00000143622	RIT1	0.030444	0.864708	up
ENSG00000163564	PYHIN1	0.030631	-1.4525	down
ENSG00000176783	RUFY1	0.031496	0.654268	up
ENSG00000107263	RAPGEF1	0.032437	-0.5992	down
ENSG00000184270	H2AC21	0.032671	-0.77486	down
ENSG00000244687	UBE2V1	0.033077	1.247281	up
ENSG00000172059	KLF11	0.033546	-2.02393	down
ENSG00000255112	CHMP1B	0.034315	1.172022	up
ENSG00000137133	HINT2	0.03444	-3.39021	down
ENSG00000215193	PEX26	0.034691	2.062326	up
ENSG00000133321	PLAAT4	0.03473	-0.65363	down
ENSG00000163932	PRKCD	0.036475	0.773031	up
ENSG00000015285	WAS	0.037959	0.60375	up
ENSG00000196313	POM121	0.038182	-1.15236	down
ENSG00000074755	ZZEF1	0.038615	-1.04987	down
ENSG00000094975	SUCO	0.039027	0.831962	up
ENSG00000197372	ZNF675	0.039596	2.043903	up
ENSG00000112365	ZBTB24	0.040781	-2.08374	down
ENSG00000100351	GRAP2	0.042412	0.631413	up
ENSG00000033050	ABCF2	0.042467	-1.0362	down
ENSG00000119314	PTBP3	0.043109	0.592799	up
ENSG00000270800	RPS10-NUDT3	0.043157	2.146459	up
ENSG00000166963	MAP1A	0.043679	-2.57825	down
ENSG00000136710	CCDC115	0.043705	1.737544	up
ENSG00000188486	H2AX	0.043761	2.00797	up
ENSG00000197157	SND1	0.043813	-0.6606	down
ENSG00000105559	PLEKHA4	0.044362	-1.51678	down
ENSG00000055130	CUL1	0.045369	-0.80061	down
ENSG00000107863	ARHGAP21	0.045763	1.343528	up
ENSG00000116221	MRPL37	0.04672	1.264366	up
ENSG00000110697	PITPNM1	0.046935	-1.26335	down
ENSG00000170921	TANC2	0.047316	2.686476	up
ENSG00000110025	SNX15	0.047671	3.333243	up
ENSG00000070182	SPTB	0.048006	0.859222	up
ENSG00000078142	PIK3C3	0.048936	0.677108	up
ENSG00000204389	HSPA1A	0.048937	1.410393	up
ENSG00000137101	CD72	0.049889	1.362187	up

### Verification of exosomal mRNA expression by qRT−PCR

**Figure 4:**
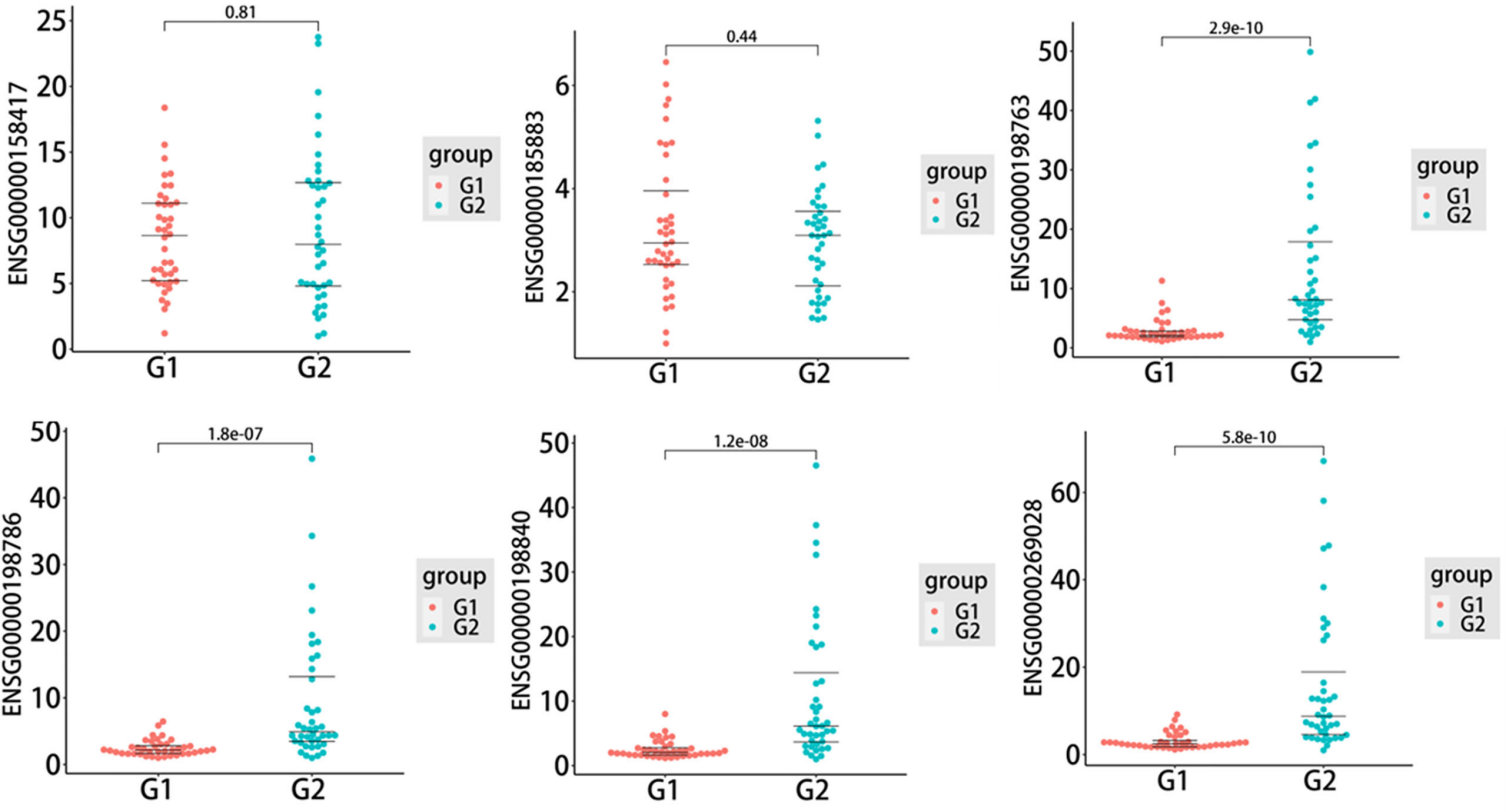
The quantitative real-time PCR (qRT−PCR) results of six selected differentially expressed mRNAs (DEMs) in the type 1 diabetes mellitus (T1DM) and control groups (T1DM N=40; controls N=40). Statistically significant differences (*P* value < 0.05) were calculated by unpaired t test. The three lines in each figure represent the median and interquartile spacing. G1: control group; G2: case group.

To further assess the biomarker potential for T1DM, six exosomal DEMs, including five upregulated DEMs, ENSG00000185883 (ATP6VOC), ENSG00000198763 (MT-ND2), ENSG00000198786 (MT-ND5), ENSG00000198840 (MT-ND3) and ENSG00000269028 (MTRNR2L12), and one downregulated DEM, ENSG00000158417 (MTRNR2L12), were selected to validate the sequencing data in an independent cohort. As shown in **Figure 4**, the qRT−PCR results indicated that four of six candidate mRNAs, namely, ENSG00000198763 (MT-ND2) (*P*=2.9e-10), ENSG00000198786 (MT-ND5) (*P*=1.8e-07), ENSG00000198840 (MT-ND3) (*P*=1.2e-08) and ENSG00000269028 (MTRNR2L12) (*P*=5.8e-10), were significantly upregulated in T1DM patients compared with control subjects, demonstrating the predictive accuracy of the sequencing data and the biomarker potential of exosomal mRNAs. The remaining two mRNAs, that is, ENSG00000185883 (ATP6VOC) and ENSG00000158417 (MTRNR2L12), failed to reach statistical significance.

### Functional analysis of exosomal mRNAs

**Figure 5:**
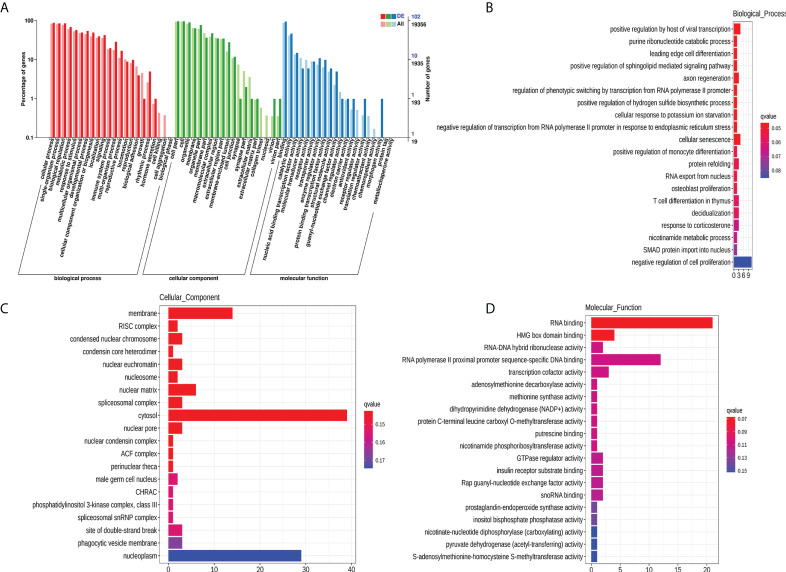
GO enrichment analysis of 112 DEMs. GO categories of DEMs **(A)**. Top 20 significantly enriched biological processes **(B)**, cellular components **(C)**, and molecular functions **(D)** of DEMs. The x-axis represents the number of mRNAs.

To explore the potential biological function of 112 DEMs, GO enrichment analysis and KEGG pathway analysis were employed. The GO database is a structured standard biological annotation system and aims to establish a standard vocabulary system of genes and their products. The GO annotation system includes three main branches, namely, biological process, molecular function, and cellular component. We summarized the significantly enriched GO terms of mRNAs with respect to biological process (**Table S3**), cellular component (**Table S4**), and molecular function (**Table S5**) and (**Figure 5A**). The mRNAs were significantly associated with biological processes, such as positive regulation by host of viral transcription (GO:0043923), axon regeneration (GO:0009154), and cellular senescence (GO:0035026) (**Figure 5B**). The top 10 GO terms (“positive regulation by host of viral transcription” to “cellular senescence”) of biological processes still showed statistical significance after correcting the *P* value (**Table S3**). Membrane (GO:0016020), RISC complex (GO:0016442), and condensed nuclear chromosome (GO:0000794) were the most enriched cellular components (**Figure 5C**). RNA binding (GO:0044822), HMG box domain binding (GO:0071837), and RNA−DNA hybrid ribonuclease activity (GO:0004523) were mostly involved in molecular function (**Figure 5D**). However, the results of the cellular component and molecular function analyses should be interpreted with caution because the *Q*-value (corrected *P* value) indicated that the significance disappeared after multiple hypothesis testing (**Tables S4**, **S5**).

**Figure 6:**
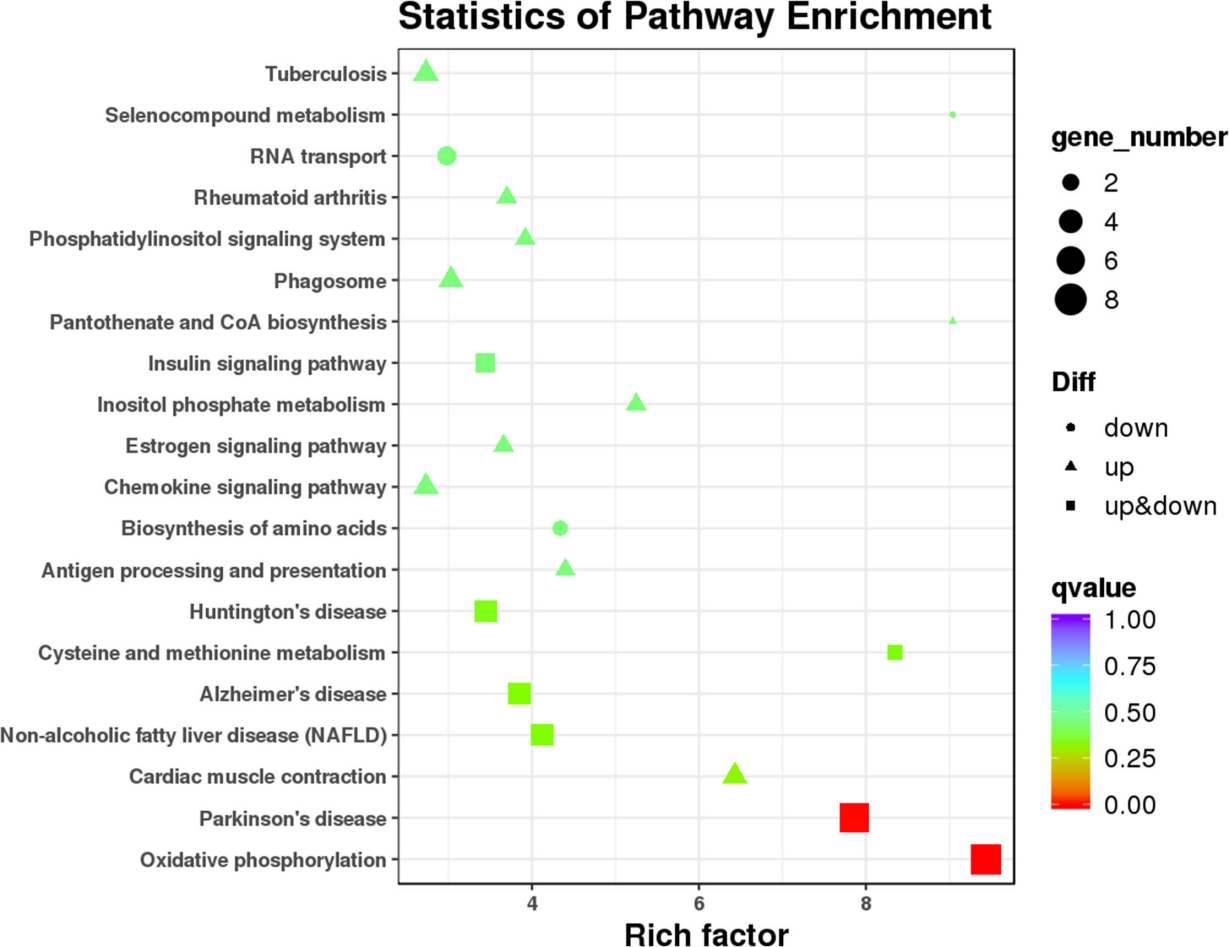
Kyoto Encyclopedia of Genes and Genomes (KEGG) pathway analysis of 112 DEMs.

As the main public database related to the pathway enrichment analysis, KEGG provides a query of the integrated metabolic pathways, including metabolism of carbohydrates, nucleosides, and amino acids and the biodegradation of organic matter. It not only provides all possible metabolic pathways but also comprehensively annotates the enzymes that catalyze each step of the reaction. In this study, KEGG pathway analysis (**Figure 6** and **Table S6**) suggested that the DEMs between T1DM patients and healthy controls involved in oxidative phosphorylation (ko00190) and Parkinson’s disease (ko05012) and other pathways did not reach statistical significance after correcting the *P* values.

### Establishment of the PPI network

**Figure 7:**
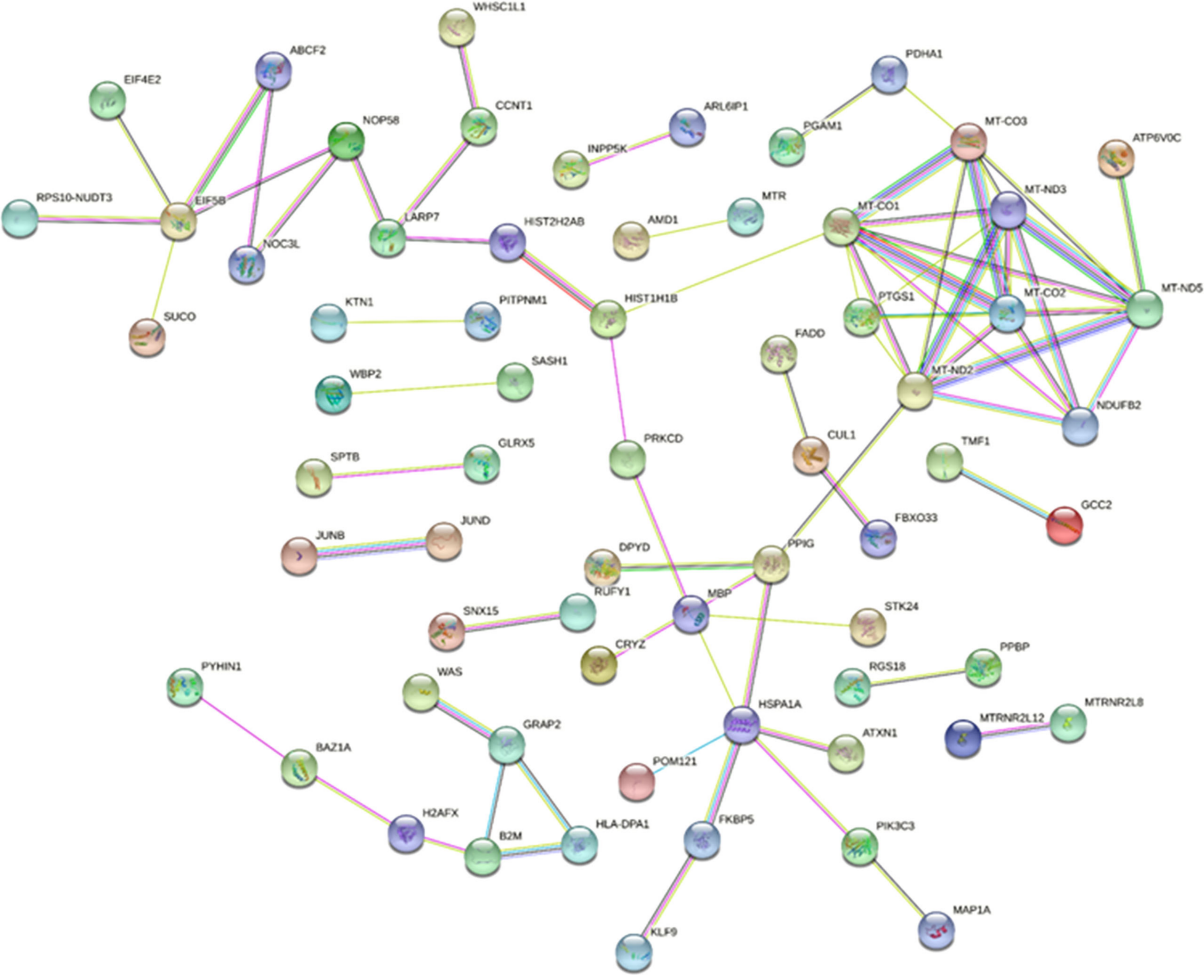
The protein−protein interaction network of DEMs. Network nodes represent proteins, and edges represent protein−protein associations.

STRING is a database of protein−protein interactions that have been predicted and experimentally verified, including direct physical interactions and indirect functional correlations, in multiple species. Combined with the results of differential expression analysis and the interaction pairs recorded in the database, the PPI network of exosomal DEMs was constructed (**Figure 7**). In total, 66 nodes and 74 protein interaction pairs were identified in the PPI network. According to the score obtained by five algorithms, 8 hub mRNAs, including MT-ND5, MT-ND2, MT-CO1, MT-ND3, MT-CO2, MT-CO3, NDUFB2, and PTGS1, were identified. Among them, MT-ND5 ranks first in each algorithm and is the core hub gene.

## Discussion

Existing studies have emphasized the importance of exosomes in multiple pathological processes (7, 16). Valadi et al. first reported that exosomes contain certain mRNAs and that exosomal mRNAs can be delivered to other cells and translated into proteins in a new location (6). Yokoi et al. reported that MMP1 mRNA-carrying extracellular vesicles from highly metastatic cells can induce apoptosis in mesothelial cells and facilitate the peritoneal dissemination of ovarian cancer (17). In addition, *in vivo* experiments have indicated that exosomes can transfer the mRNA encoding Cre recombinase and can induce Cre-LoxP-mediated recombination in recipient cells (18). These results suggested that exosomes can lead to the delivery of functional mRNA and can alter the phenotype of recipient cells. However, research concerning the association between T1DM and exosomal mRNAs is relatively lacking. In this study, we characterized the expression profiles of exosomal mRNAs in the plasma of T1DM patients and further explored the biomarker potential of the identified exosomal mRNAs. In addition, we performed bioinformatic analysis (GO and KEGG) to clarify the biological functions of the identified DEMs.

Here, a total of 112 exosomal DEMs were detected, of which 66 mRNAs were upregulated and 46 mRNAs were downregulated in T1DM patients compared to controls. These results strongly indicated that the expression profiles of plasma-derived exosomal mRNAs in patients with T1DM differ from those in healthy individuals. Next, six candidate mRNAs were selected to verify the sequencing results by qRT−PCR. Four of six mRNAs, namely, ENSG00000198763 (MT-ND2), ENSG00000198786 (MT-ND5), ENSG00000198840 (MT-ND3) and ENSG00000269028 (MTRNR2L12), were indicated to have significant differences between T1DM patients and control subjects and followed the same tendency as the sequencing data. This result highlighted that plasma-derived exosomal mRNAs might serve as novel minimally invasive diagnostic tools for T1DM. However, two of six mRNAs failed to show statistical significance in the qRT−PCR analysis. Therefore, the biomarker use of other DEMs identified by sequencing requires further validation. At present, the lack of a robust biomarker for T1DM prevents the early recognition and intervention that could preserve residual pancreatic beta-cells. Previous studies have strongly suggested the possible biomarker utility of plasma RNA (19, 20). However, there are some alternative advantages with regard to exosomal RNA in disease diagnosis. For instance, exosomal RNAs tend to be more stable in plasma because the lipid bilayer-enclosed structure can protect the RNA cargo from degradation. In addition, the release and contents of exosomes are strictly regulated by physical and pathological conditions, thus reflecting the biological status of the body.

Next, we performed bioinformatic analysis to predict the biological functions of the identified exosomal DEMs. GO enrichment analysis revealed that the most significantly enriched term was positive regulation by host of viral transcription among the biological processes. Previous studies have also indicated that viral infections play an essential role in T1DM development. Data revealed that more T1DM patients were diagnosed during cold months (21, 22), which suggested that viruses play a role in triggering T1DM because viral infections are more common in winter than in summer. Viral respiratory tract infections in early life have also been reported to be associated with islet autoimmunity and beta-cell damage (23, 24). Moreover, some viruses, such as coxsackieviruses, can induce the onset of T1DM in animal models and can be isolated from the pancreas in patients with newly diagnosed T1DM (25–27). In addition to pathogenic effects, some studies have also shown the protective role of viral infection, which is in agreement with the hygiene hypothesis. It has been reported that the inoculation of young nonobese diabetic (NOD) mice, models of spontaneous autoimmune diabetes mellitus, with some viruses was followed by long-term protection from T1DM (28, 29). However, the underlying mechanisms behind the interactions of viral infections and the onset of T1DM have not been fully elucidated. Our results suggested that exosomal mRNAs might be involved in this process, but the exact functions of the exosomal mRNAs need further investigation. KEGG analysis indicated that the main pathways of the identified DEMs were involved in oxidative phosphorylation and Parkinson’s disease. Interestingly, our previous study also indicated that the KEGG pathway analysis regarding exosomal lncRNAs was associated with Parkinson’s disease (13). Oxidative phosphorylation (OXPHOS) is a metabolic pathway with low glucose utilization and high ketone production. Generally, immune cells generate energy through OXPHOS or aerobic glycolysis, another metabolic pathway associated with high glucose utilization. However, it has been indicated that aerobic glycolysis is suppressed while OXPHOS is exacerbated in individuals with T1DM (30, 31). The dysregulation of lymphoid metabolism might partially explain why T1DM patients were more susceptible to ketosis than type 2 diabetes mellitus (T2DM) patients (32). More interestingly, it has been indicated that the bacillus Calmette-Guerin (BCG) could lower blood glucose levels by shifting the glucose metabolism of the immune system from overactive OXPHOS to aerobic glycolysis (30, 31). Taken together, the bioinformatic analysis suggested that these identified DEMs were involved in the development of T1DM *via* different pathways. In addition, we constructed a PPI network and identified 8 hub genes (MT-ND5, MT-ND2, MT-CO1, MT-ND3, MT-CO2, MT-CO3, NDUFB2, and PTGS1) among the identified exosomal DEMs. These genes might play an important role in T1DM. For example, a recent study indicated that rare variants within MT-ND5 were associated with fasting insulin (33). In addition, it has been indicated that MT-ND2 could modify resistance against T1DM in NOD mice by modulating beta-cell sensitivity to T-cell effectors (34). However, the exact role of these genes in T1DM in the context of exosomes needs further investigation.

In conclusion, this study identified the characteristics of the plasma-derived exosomal mRNA transcriptome of T1DM for the first time, and the results highlighted the biomarker potential of exosomal mRNA.

## Data availability statement

The datasets presented in this study can be found in online repositories. The names of the repository/repositories and accession number(s) can be found below: https://db.cngb.org/search/project/CNP0002574/, CNP0002574.

## Ethics statement

The studies involving human participants were reviewed and approved by the institutional ethics review board of the Second Xiangya Hospital of Central South University. The patients/participants provided their written informed consent to participate in this study.

## Author contributions

ZZ, ZX, and HP conceived and designed the experiments. WF, HP, JiaqL, YW, SL, JianL, HY, YX, GH, and XL collected samples. WF, HP, and XS performed the experiments and analyzed the data. WF and HP wrote the manuscript. All authors contributed to the article and approved the submitted version.

## Funding

This work was supported by the National Key R&D Program of China (grant number 2018YFE0114500), the National Natural Science Foundation of China (grant numbers 82070813 and 81873634), and the Hunan Province Natural Science Foundation of China (grant numbers 2022JJ30858, 2018JJ2573, and 2020JJ2053).

## Conflict of interest

The authors declare that the research was conducted in the absence of any commercial or financial relationships that could be construed as a potential conflict of interest.

## Publisher’s note

All claims expressed in this article are solely those of the authors and do not necessarily represent those of their affiliated organizations, or those of the publisher, the editors and the reviewers. Any product that may be evaluated in this article, or claim that may be made by its manufacturer, is not guaranteed or endorsed by the publisher.
